# N-Terminal protein sequencing by nicotinic acid derivatization and MS analysis

**DOI:** 10.1007/s00216-026-06727-4

**Published:** 2026-08-11

**Authors:** Peter Gehrig, Simone Wüthrich, Serge Chesnov, Ralph Schlapbach, Paolo Nanni

**Affiliations:** Functional Genomics Center Zurich, University of Zurich/ETH Zurich, Zurich, Switzerland

**Keywords:** N-Terminomics, N-Terminal, Protein sequencing, Dimethylation, Nicotinic acid

## Abstract

Most MS-based workflows for N-terminal protein sequence analysis rely on the modification of primary amines at the protein N-terminus and lysine residues, in many cases through dimethylation. However, incomplete dimethyl labeling and undesired side reactions have been reported previously and were also observed in our experiments. In this study, we evaluated alternative methods for N-terminal protein derivatization. For reductive dimethylation, the highly toxic reducing agent sodium cyanoborohydride was successfully replaced by pyridine borane. In addition, several single-reagent labeling methods were applied to model proteins and compared to the dimethylation workflow. Criteria for the selection of suitable reagents were completeness and selectivity of the protein derivatization reaction, the detection sensitivity for modified peptides by mass spectrometry, and the absence of undesirable side reactions. Comparative analyses demonstrated that the chemical derivatization of free amines by nicotinic acid N-hydroxysuccinimide ester is a particularly promising approach for N-terminal protein sequencing. Protocols for the nicotinoylation of proteins in solution or in gel using this reagent were optimized and successfully applied to various test proteins.

**Graphical Abstract:**

**Figure:**
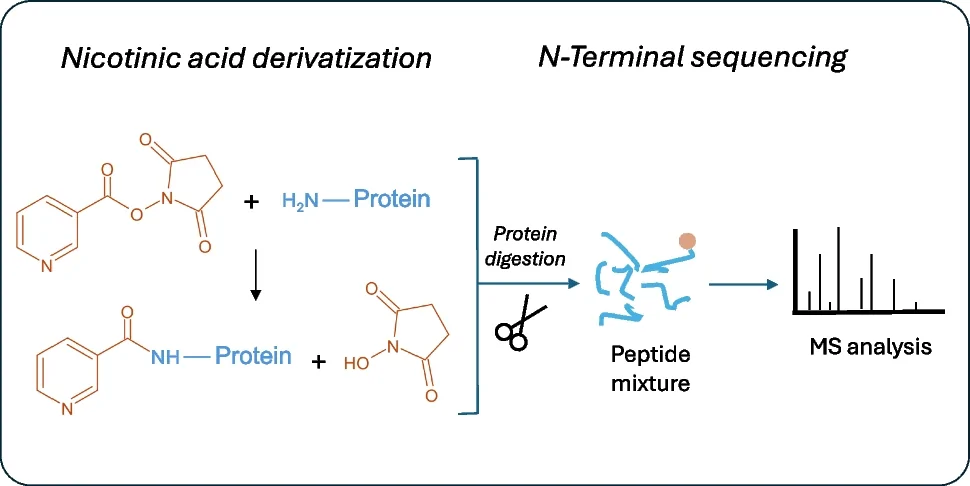

## Introduction

MS-based analysis of N-terminal protein sequences permits reliable identification of real N-terminal ends of mature proteins and of protease substrates. Genetic information encoding for protein sequences is not sufficient to identify different protein products from a single gene, for example multiple proteoforms resulting from alternative RNA splicing or alternative translation initiation. Moreover, protein N-termini are often co- or posttranslationally modified. The most common modifications occurring at protein N-termini include removal of the initiator methionine or of signal and propeptides, processing by aminopeptidases, acetylation, myristoylation, or other acyl modifications, and pyroglutamate formation [1–3]. These proteolytic processing events and post-translational modifications may regulate the activity, lifetime, and localization of proteins, and even interactions with other proteins.

The classical method for N-terminal sequencing, Edman degradation [4], has been largely replaced by various mass spectrometric methods [1, 3, 5, 6], which usually allow identification not only of free, but also of modified protein N-termini. N-terminal sequencing of purified proteins in solution or in gel bands is often requested in protein analysis facilities, for example to characterize real N-termini of intact proteins or to identify new N-termini of product-related fragments [7, 8]. In addition, workflows have been developed for proteome-wide N-terminome analysis [9, 10], which typically include an enrichment step for N-terminal peptides. An important and widespread application of N-terminal protein sequencing methods are investigations of protease substrates and cleavage products. Proteases are involved in many biological processes, but the identification of their substrates and cleavage sites remains challenging.

An essential step of most MS-based N-terminal sequencing workflows is the chemical derivatization of the N-terminal amino group. In most cases, the amino groups of lysine side chains are derivatized, too. Dimethyl labeling of protein amino groups, also known as reductive methylation, has been widely used for N-terminal protein sequencing. Dimethylation in a mixture of formaldehyde and sodium cyanoborohydride as reducing agent is a relatively efficient and straightforward procedure and has been established as a standard method for this purpose [9, 11–15]. In addition, isotopic labeling of peptides by reductive methylation of the N-termini and of lysine side chains is utilized for quantitative proteomic analysis [16–19]. However, it is unclear if complete derivatization of amino groups by this method has ever been achieved [20], and various undesirable side reactions have been reported [21–24]. Moreover, formaldehyde and particularly the reducing agent sodium cyanoborohydride are known as hazardous chemicals.

In the present study, alternative chemical reagents for N-terminal modification of proteins were introduced and applied to model proteins to evaluate efficiency and selectivity of the labeling reactions. For the reductive methylation method, the commonly applied reagent sodium cyanoborohydride was replaced by pyridine borane, which had been proposed as an alternative reducing agent already in 1982 [25, 26]. In addition, several derivatization methods requiring only one reagent were evaluated and compared to the dimethyl labeling approach. These reagents included RapiFluor-MS (RapiFluor), a reagent containing an amine-reactive N-hydroxysuccinimide (NHS) carbamate group [8, 27], and 2-pyridinecarboxaldehyde (2-PCA), which specifically reacts with protein N-termini, but not with lysine residues [28]. Furthermore, we evaluated tandem mass tag (TMT) reagents [29] and nicotinic acid NHS ester, which contain reactive NHS ester groups. Nicotinic acid NHS ester had been applied previously to enhance de novo sequencing of peptides [30] and for quantitative proteomics experiments [30, 31], but not for N-terminal sequencing of proteins, to our knowledge. Based on chemical criteria and on comparative analyses of tandem mass spectra of modified peptides, we selected the nicotinic acid NHS ester derivatization method for all following experiments. Protocols for proteins in solution or in gel pieces were optimized, particularly in view of better modification yields and fewer side reactions.

## Methods

### Materials

Myoglobin (horse), bovine serum albumin (BSA), β-lactoglobulin, α-casein, and β-galactosidase (*E. coli*) were purchased from Sigma-Aldrich. Trypsin (sequencing grade, frozen) and recombinant chymotrypsin (rChymoSelect) were purchased from Promega, and purified chymotrypsin (sequencing grade) was purchased from Roche. Buffer solutions containing 2-morpholinoethanesulfonic acid (MES, 0.5 M, pH 6.5) or sodium phosphate (0.5 M, pH 7.0, 7.5, or 8.0) were purchased from Alfa Aesar. Triethylammonium bicarbonate solution (TEAB, 1 M, pH 8.5), ammonium hydroxide solution (28–30%), ammonium bicarbonate, sodium cyanoborohydride, pyridine borane (8 M borane in pyridine), and 2-pyridinecarboxaldehyde (2-PCA) were purchased from Sigma-Aldrich. 4-(2-hydroxyethyl)−1-piperazineethanesulfonic acid buffer solution (HEPES, 0.5 M, pH 7.5), tris(2-carboxyethyl) phosphine hydrochloride (TCEP), hydroxylamine solution (50%), formaldehyde (16% (w/v) in water), tandem mass tag label reagents (TMT2-126 and TMT2-127), and NuPAGE 4–12% Bis-Tris gels (1 mm) were purchased from Thermo Fisher Scientific. GlycoWorks RapiFluor was obtained from Waters, and nicotinic acid NHS ester was obtained from Toronto Research Chemicals.

### Chemical derivatization of proteins in solution

#### Dimethylation

25 µg of protein in 200 mM HEPES buffer (pH 7.5) was dimethylated in a mixture of 50 mM formaldehyde and 25 mM sodium cyanoborohydride or 50 mM pyridine borane for 2 h at room temperature (25 °C). Pyridine borane was diluted 1:8 with ethanol and added to the aqueous reaction solution, resulting in a final ethanol concentration of 5% (v/v). In the optimized protocol, TCEP was added to a concentration of 4 mM, and guanidine HCl to a concentration of 4 M. The concentration of formaldehyde was 50 mM as before, and the concentration of pyridine borane was 25 mM or 50 mM.

#### RapiFluor derivatization

 15–40 μg of protein in 30 µl of 200 mM phosphate buffer (pH 6.5) was labeled using RapiFluor in reagent to protein ratios of 20:1 or 60:1 (w/w) at room temperature for 30 min. The reagent was dissolved in N-dimethylformamide at a concentration of 68 μg/μl following the instructions of the vendor.

#### 2-PCA derivatization

25 μg of protein in 200 mM HEPES buffer (pH 7.5) was incubated with 20 mM 2-PCA and 5 mM TCEP for 3 h at 37 °C.

#### TMTduplex labeling

25 μg of protein in 45 µl of 50 mM TEAB solution (pH 8.5) was labeled with 100 µg TMT2-126 or TMT2-127 reagent dissolved in 15 µl acetonitrile (ACN) for 1 h at room temperature. The reagent was quenched with hydroxylamine (0.3%, v/v) for at least 15 min at room temperature. 

#### Nicotinic acid modification

According to our initial protocol, 25 µg protein in 200 mM HEPES buffer (pH 7.5) was labeled with 10–20 mM nicotinic acid NHS ester. The reagent, which is not well soluble in purely aqueous or organic solvents, was first dissolved in 65% ethanol and then added to the reaction solution. The reaction was allowed to proceed for 2 h at room temperature (25 °C), and the reagent was quenched with 1% hydroxylamine at room temperature for 30 min. The following buffer solutions were tested to optimize the reaction yield in the pH range from 6.5 to 8.5: MES (pH 6.5), sodium phosphate (pH 7.0, 7.5, and 8.0), HEPES (pH 7.5), and TEAB (pH 8.5).

In our optimized protocol, proteins were dissolved in HEPES buffer pH 7.5 (100 mM final concentration) containing 4 mM TCEP and 4 M guanidine hydrochloride. A stock solution of 100 mM nicotinic acid NHS ester in 65% ethanol was prepared and added to a concentration of 20 mM in the reaction solution, and the reaction time at room temperature was 2 h. To quench the reagent, 1% of hydroxylamine was added, the pH was increased to 10 by the addition of 1% ammonium hydroxide, and the reaction was allowed to proceed for 1 h at room temperature.

### Nicotinic acid NHS ester derivatization of proteins in gel

Proteins were separated in NuPAGE 4–12% Bis-Tris gels (1 mm; Thermo Fisher Scientific) according to the protocol of the vendor. Gel bands were excised and cut into smaller pieces. The gel pieces were destained twice with 50% ACN for 15 min at 50 °C and dehydrated in 80% ACN at room temperature for approximately 5 min, until they turned white. The nicotinoylation reaction was conducted in 100 mM HEPES buffer (pH 7.5) containing 4 mM TCEP. A 200 mM stock solution of nicotinic acid NHS ester in 65% of ethanol was prepared and added to a final concentration of 40 mM reagent in the reaction solution. The reaction was allowed to proceed for 2 h at room temperature. Next, the reaction solution was removed, and the gel pieces were dried with 80% acetonitrile. The reagent was inactivated by the addition of 1% hydroxylamine and 1% ammonium hydroxide in 100 mM HEPES buffer at pH 10 and at room temperature during 1 h.

### Proteolytic digestion of proteins in solution

Proteins labeled with RapiFluor or TMT reagents were proteolytically digested by using the Filter Aided Sample Preparation (FASP) method [32]. Protein samples were transferred to 10 kDa Microcon centrifugal filters (Sigma-Aldrich) and washed twice with 200 µl of 100 mM TEAB solution (pH 8.5). Subsequently, the proteins were digested with 2–3% of trypsin or with 4% of chymotrypsin in 100 ul of TEAB solution at room temperature overnight.

All other protein samples were cleaved by using the single-pot, solid-phase-enhanced sample-preparation (SP3) protocol [33]. Hydrophilic and hydrophobic carboxylate-modified magnetic beads (GE Life Sciences) were mixed in a 1:1 ratio and washed with water twice. Ethanol was added to myoglobin and α-casein samples to a final volume of 80% (v/v) and to the other protein samples to a volume of 70% (v/v). Washed SP3 beads were mixed with proteins in a 1:10 ratio (w/w) protein/beads. After 30 min incubation at room temperature, the supernatant was removed, followed by two wash steps with 80% ethanol. 2% of trypsin or 2–4% of chymotrypsin was added, and the proteolytic digestion on SP3 beads was conducted in 100 μl of 100 mM TEAB solution overnight, typically at room temperature. The digestion of 2-PCA-labeled proteins was limited to 4 h, to minimize partial loss of the modification.

### Proteolytic digestion of proteins in gel

The gel pieces were washed with 50% ACN twice. 200 ng of trypsin, 400 ng of purified chymotrypsin, or 400 ng of rChymoSelect in 10 µl of 100 mM TEAB buffer was added. The gel pieces were covered by the addition of 20–30 μl of TEAB buffer, and the proteolytic digestion was allowed to proceed at room temperature overnight. The supernatant was collected, and 30 µl 50% ACN containing 0.1% TFA were added to the gel pieces to extract peptides more completely. The combined supernatants were dried prior to LC-MS analysis.

### LC-MS/MS analysis

Data-dependent acquisition (DDA) analyses were performed on an Orbitrap Fusion Lumos Tribrid mass spectrometer (comparisons of different protein derivatization methods) or on a Q Exactive Orbitrap mass spectrometer (Thermo Fisher Scientific; optimization and validation of nicotinoylation protocols). Both instruments were equipped with a Digital PicoView ion source (New Objective, Woburn, MA) and coupled to an AQUITY UPLC M-class system (Waters). Liquid chromatography solvent compositions of buffers A and B were 0.1% formic acid in water and 0.1% formic acid in ACN, respectively. The samples were either diluted with or re-dissolved in 3% ACN containing 0.1% formic acid. Sample solutions were loaded onto a Symmetry C18 trap column (100 Å, 5 µm, 180 µm × 20 mm, Waters) and separated on a HSS T3 analytical column (100 Å, 1.8 µm, 75 µm × 250 mm, Waters). Peptides were eluted at a flow rate of 300 nL/min.

For the Orbitrap Fusion Lumos analyses, the following LC gradient was applied: 0–3 min: 5% buffer B, 43 min: 25% B, 53 min: 60% B, 63 min: 95% B. Survey scans were acquired in the Orbitrap mass analyzer in the range of m/z 300–2000, with a resolution of 120,000, an automated gain control (AGC) target value of 400,000, and a maximum injection time of 50 ms. Higher energy collisional dissociation (HCD) spectra were acquired in the Orbitrap mass analyzer, using a normalized collision energy of 33%, a resolution of 60,000, an AGC target value of 50,000, and a maximum injection time of 118 ms. Precursor ions were isolated in the quadrupole with an m/z 1.6 isolation window. Charge state screening was enabled, and only precursor ions with charge states 2–6 were included. The threshold value for signal intensities was 25,000. Precursor ion masses already selected for MS/MS acquisition were dynamically excluded for 30 s.

The following LC gradient was used for the Q Exactive Orbitrap analyses: 0–3 min: 3% buffer B, 45 min: 35% B, 50 min: 60% B, 60 min: 95% B. Survey scans were acquired in the range of m/z 300–1500, with a resolution of 70,000, an AGC target value of 3,000,000, and a maximum injection time of 100 ms. HCD spectra were acquired by using normalized collision energy of 25%, a resolution of 35,000, an AGC target value of 100,000, and a maximum injection time of 120 ms. Precursor ions were isolated in the quadrupole with an m/z 1.6 isolation window. Charge state screening was enabled, and precursor ions with charge state 1 or unassigned charge states were excluded. The 12 most intense precursor ions per full scan were selected for MS/MS acquisition. The threshold value for signal intensities was 25,000. Precursor ion masses already selected for MS/MS acquisition were dynamically excluded for 10 s.

The data were handled using the local laboratory information management system (LIMS) [34]. The mass spectrometry proteomics data have been deposited to the ProteomeXchange Consortium via the PRIDE (http://www.ebi.ac.uk/pride) partner repository with the dataset identifier PXD081064.

### Data analysis

MS and MS/MS spectra were converted to Mascot generic format (MGF) files by using Proteome Discoverer, version 2.1 (Thermo Fisher Scientific). The MGF files were searched against the UniProtKB bovine protein (taxonomy: 9913, 32,405 sequences), horse protein (taxonomy: 9796, 28,093 sequences), or *E. coli* protein database (taxonomy: 83334, 5568 sequences). The Mascot search engine (Matrix Science, London, UK; version 3.0.0) was used for peptide and protein sequence identification [35]. Mascot searches were performed with a parent ion mass tolerance of 7 ppm and a fragment ion mass tolerance of 10 ppm. Typically, a target false discovery rate of 1% was selected, and the results were refined with machine learning using Percolator. Enzyme specificity was set to semi-specific trypsin, allowing up to three missed cleavages, or semi-specific chymotrypsin, allowing up to four missed cleavages. Cleavages at phenylalanine, tyrosine, tryptophan, leucine, and methionine were considered as specific for purified chymotrypsin (Roche), and cleavages at phenylalanine, tyrosine, and leucine as specific for recombinant chymotrypsin (Promega). Oxidation of methionine, N-terminal acetylation of proteins, and the chemical modifications of interest were specified as variable modifications on N-terminal amino acids and on lysine residues. The chemical modifications included mono- and dimethylation, nicotinoylation (mass increase of 105.0215 Da), and reaction products with RapiFluor (+ 312.1586 Da), 2-PCA (+ 89.0265 Da or + 107.0311 Da), and TMTduplex (+ 225.1558 Da) reagents. For the detection of side reactions of TMT and nicotinic acid NHS ester reagents, serine, threonine, and tyrosine were considered as potential acceptor sites. The following side reaction products of amino groups induced by the treatment with formaldehyde and sodium cyanoborohydride were included as variable modifications: Methylol (+ 30.0106 Da), methylol and methylation, cyanomethylation (+ 39.0109 Da), cyanomethylation and methylation, N-terminal cyclization product (N-methyl-4-imidazolidinone, + 12.0000 Da) without and with additional methylation.

## Results and discussion

### Protein dimethylation

**Fig. 1:**
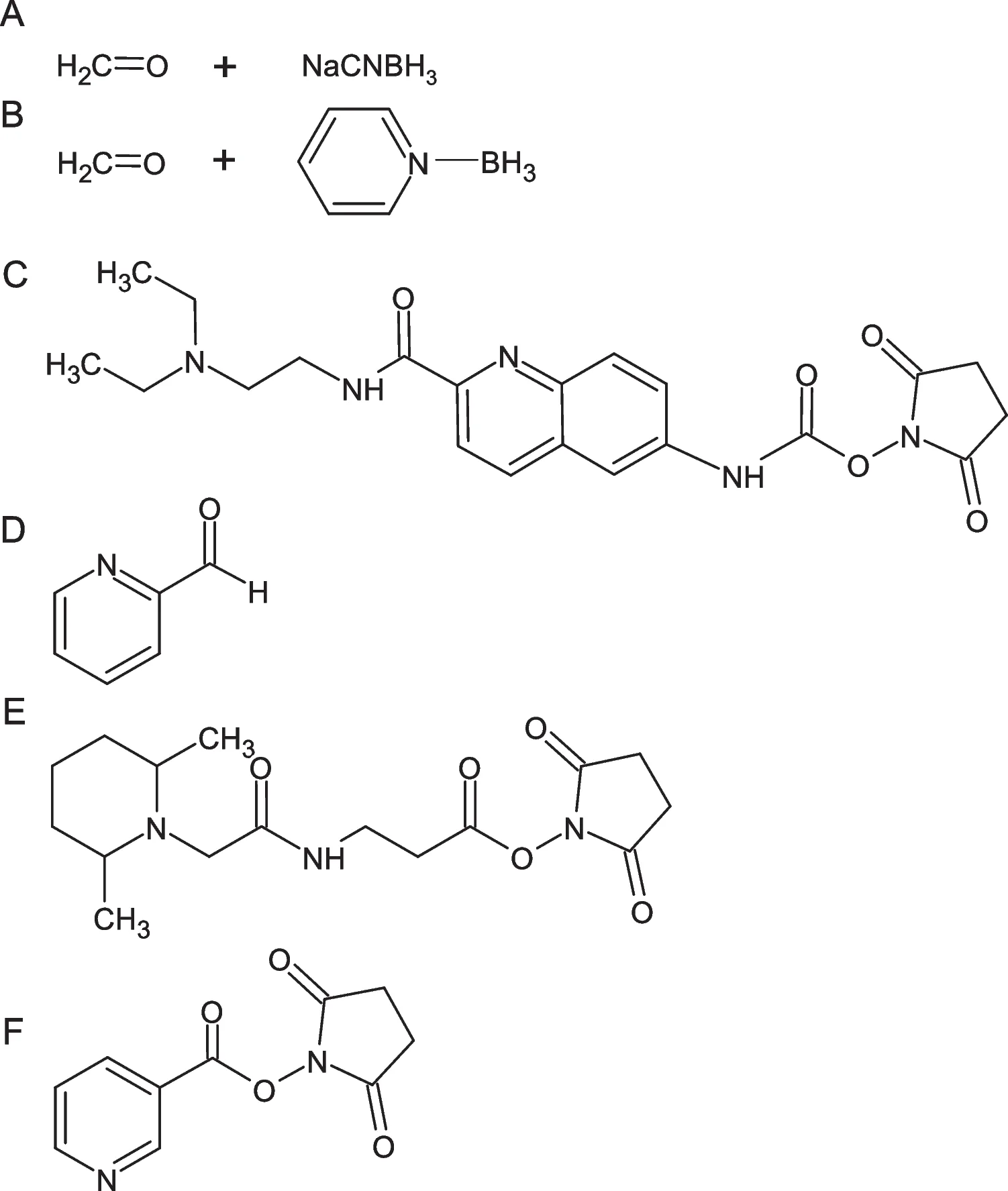
Chemical structures of reagents used for N-terminal protein labeling. **A** Formaldehyde and sodium cyanoborohydride. **B** Formaldehyde and pyridine borane. **C** RapiFluor. **D** 2-PCA. **E** TMT reagent. **F** Nicotinic acid NHS ester

In the initial experiments, we performed protein dimethylation in the presence of formaldehyde and one of two different reducing agents. The commonly used reducing agent sodium cyanoborohydride was compared to pyridine borane. The chemical structures of all protein derivatization reagents used in this study are displayed in Fig. 1. Bovine serum albumin (BSA) was utilized as the main test protein for dimethylation reactions and for other protein derivatization methods. Following the protein labeling reaction, the reagents were quenched, and the proteins were digested by trypsin or chymotrypsin and subjected to LC-MS/MS analysis. Labeling reactions yields were quantified based on the intensity levels of extracted ion chromatograms for modified and unmodified peptides. The reaction yields for complete dimethylation of the N-terminal BSA peptide, which contains a lysine residue, were 90% with sodium cyanoborohydride and 96% with pyridine borane as reducing agents. The comparable or even higher efficiency of reductive methylation with pyridine borane is consistent with previous studies [25, 26, 36, 37]. We identified not only dimethylated and undermethylated forms of BSA peptides, but also a variety of side products induced by formaldehyde and sodium cyanoborohydride. Incomplete reactions and side reactions previously reported in the literature include the formation of relatively stable methylol, resulting from the initial reaction of amines with formaldehyde [22], and succeeding reactions of the unstable imine intermediates. The cyclization of N-terminal imines of peptides is an often-overlooked side reaction [22, 23]. Furthermore, imine intermediates undergo cross-linking reactions with several nucleophilic amino acids [22, 24]. Even formation of N-cyanomethyl instead of N-methyl groups in the presence of formaldehyde and cyanoborohydride was observed [21]. All these undesirable side reaction products, apart from cross-linked peptides, were identified and quantified by LC-MS analysis in the present study (Table 1).

**Table 1:** Reaction products on the N-terminal BSA peptide, DTHKSEIAHR, induced by formaldehyde and sodium cyanoborohydride. Relative amounts of modified peptides were estimated from extracted ion chromatograms

Type of modification	Relative amount (%)	Mass increase (Da) per modified amine
Dimethylation	78.7	28
Methylation	8.0	14
No methylation	0.7	0
Methylol	1.5	30
Methylol, methylation	7.9	44
N-terminal cyclization product	0.4	12
N-terminal cyclization, methylation	2.0	26
Cyanomethylation	0.1	39
Cyanomethylation, methylation	0.7	53

Not only the N-terminal amine, but also the amine of the lysine residue may carry modifications. Furthermore, we detected internal peptides, which were mono- or dimethylated at the N-terminal amine, and must have formed due to residual reagents during proteolytic digestion. These modified peptides may lead to incorrect assignments of protein N-termini.

### Selection of new protein derivatization methods

The elimination of undesired reactions of internal peptides, which may cause false-positive results, was a primary objective of this study. To establish methods for N-terminal sequencing of purified proteins in solution or in gel, we decided to evaluate alternative reagents instead of optimizing the dimethylation reaction. A large variety of chemical methods for N-terminal derivatization of proteins have been published [1, 3, 5, 6]. For our preliminary test experiments, we selected suitable reagents according to the following criteria: (i) only commercially available chemical reagents were considered; (ii) the respective protein derivatization protocols should be easily applicable, straightforward, and reliable; (iii) nearly complete and specific main reactions and the absence of significant side reactions are essential. Chemical labeling of N-terminal peptides should result in comparable or better mass spectrometric sensitivity of detection, as compared to the unmodified peptides. Derivatized peptides should still contain a basic site at the N-terminal end, to enable protonation in the gas phase and efficient ionization during MS analysis. Dimethylation of peptides and proteins retains the basicity of free amino groups. All other labeling reagents utilized in this study (Fig. 1) eliminate basic sites by their reactions with amino groups, but they contain either a basic tertiary amine (RapiFluor and TMT reagents) or a pyridine ring (2-PCA and nicotinic acid).

### Preferential or selective labeling of the protein N-terminus

Most reagents used for N-terminal sequence analysis modify not only N-terminal amines but also amines of lysine side chains. However, derivatization of lysine residues largely prevents cleavage by trypsin, and a large percentage of peptides generated by trypsin cleavage only at arginine residues are too long and hydrophobic for detection by LC-MS/MS analysis. A possible solution to this problem is the utilization of complementary proteases, for example chymotrypsin. Another option is a strategy based on reagents, which mainly react with protein N-termini, but not with lysine residues. For this purpose, we performed test experiments using RapiFluor [27] or 2-pyridinecarboxaldehyde (2-PCA) [28], to preferentially or specifically label amino groups of protein N-termini (Fig. 1C and D). Myoglobin, β-lactoglobulin, and BSA were utilized as model proteins for this group of reagents, which allow trypsin cleavage at free lysine residues near the protein N-terminus.

RapiFluor molecules consist of a reactive NHS carbamate group, a quinoline fluorophore, and a basic tertiary amine for improving MS ionization. Originally, RapiFluor was developed for combined fluorescence detection and mass spectrometry analysis of released glycans [27], and the same reagent was later utilized for identification and sequencing of N-terminal peptides by using fluorescence and mass spectrometry data in one experiment [8]. A protocol for preferential labeling of proteins at the N-terminus at pH 6.5 was developed for this approach, allowing detection of N-terminal peptides by fluorescence. Our preliminary protein labeling experiments using RapiFluor demonstrated that preferential modification of N-termini could be achieved. However, the reaction yields of protein N-termini and of lysine side chains were variable and dependent on the proteins (data not shown).

**Fig. 2:**
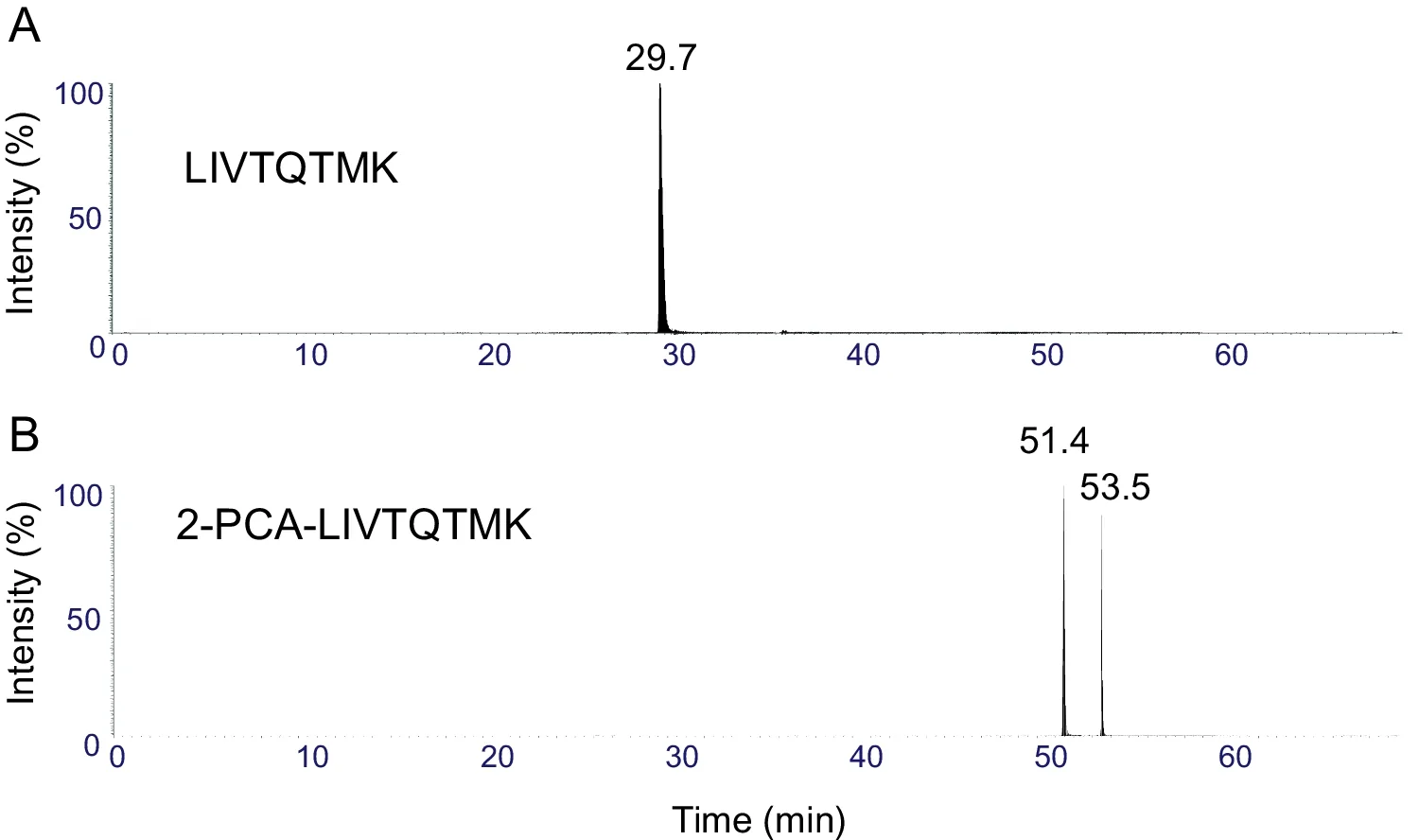
Extracted ion chromatograms of the underivatized **A** and of the 2-PCA-labeled **B** N-terminal peptide of β-lactoglobulin (LIVTQTMK). The respective doubly charged peptide ions were selected

Next, we performed test experiments using 2-PCA (Fig. 1D), which selectively reacts with free N-terminal amines of peptides and proteins [28, 38, 39]. The imines formed in the first step are stabilized by a unique cyclization reaction with the next amide nitrogen in the peptide chain, unless the second amino acid is proline. Reactions of 2-PCA with lysine residues or with other amino acids are reversible and do not lead to stable reaction products. Our test experiments confirmed that 2-PCA specifically reacted with protein N-termini, almost without side reactions with lysine or other amino acids. Unfortunately, the reactions of protein N-termini with 2-PCA tend to be inefficient, incomplete, and even partially reversible [28, 39]. Typically, the extent of 2-PCA modification in our test experiments with myoglobin, β-lactoglobulin, and BSA was in the range of 50–70% (data not shown). Furthermore, we observed that two isobaric forms of 2-PCA-labeled peptides were chromatographically separated, most likely the two diastereomers. The extracted ion chromatograms show that the unmodified N-terminal peptide of β-lactoglobulin eluted as a single peak after 30 min, while the peptide modified with 2-PCA eluted in two distinct peaks of similar intensity after 51.5 min and 53.5 min (Fig. 2).

Recently, Song et al. discovered that 2-PCA can undergo an aldol condensation with protein N-termini as a side reaction, resulting in a higher mass shift of 107 Da compared to that of the expected modification (+ 89 Da) [40]. This side reaction preferentially occurs at N-terminal glycine. Indeed, we detected both modifications as reaction products of myoglobin, which contains an N-terminal glycine residue.

**Fig. 3:**
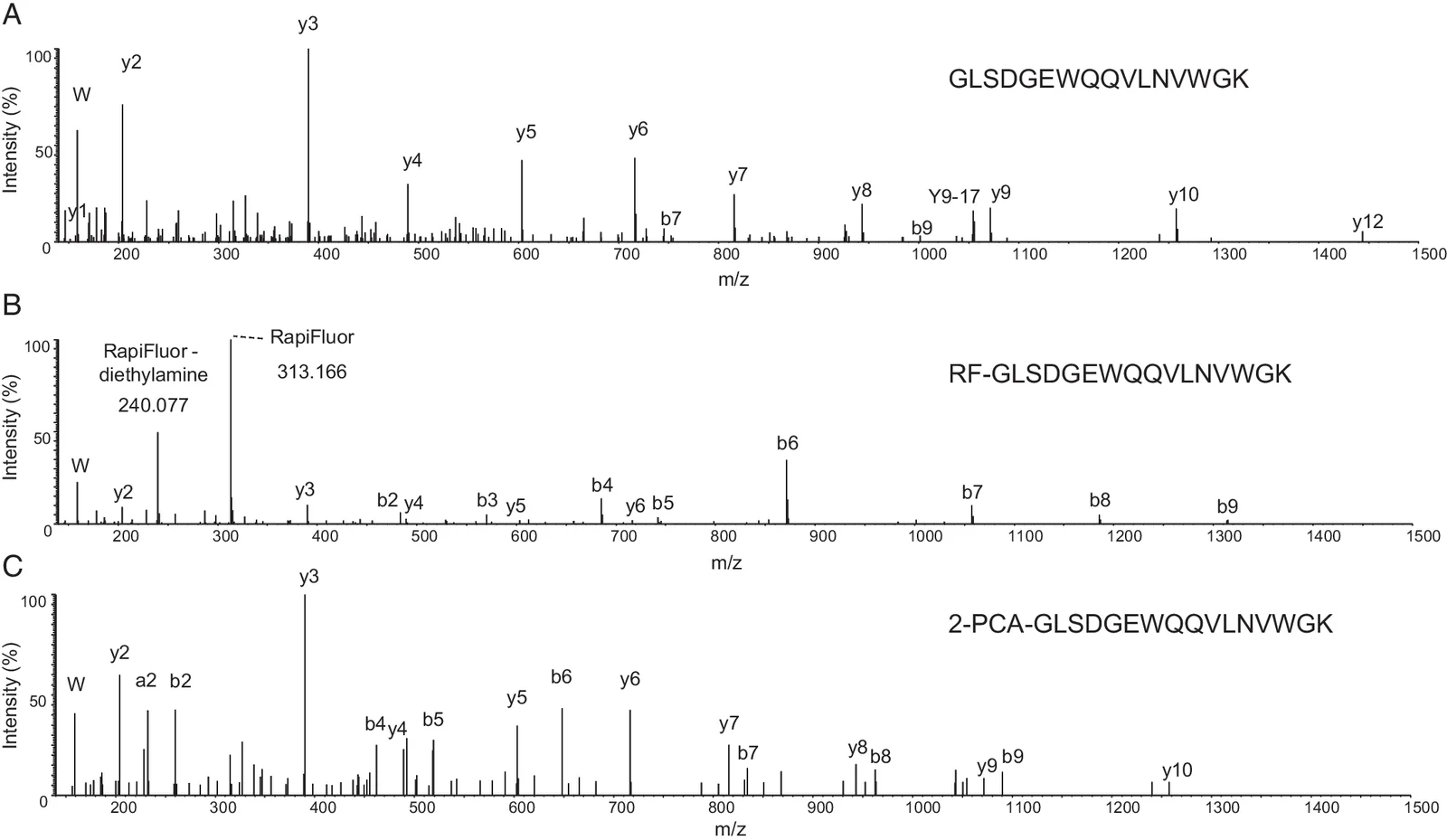
MS/MS fragmentation patterns of the N-terminal peptide of myoglobin (GLSDGEWQQVLNVWGK) **A** unmodified, **B** labeled with RapiFluor, and **C** labeled with 2-PCA

To evaluate the influence of N-terminal RapiFluor or 2-PCA labeling on the gas-phase fragmentation of peptides, we acquired MS/MS data from the unmodified N-terminal peptide of myoglobin (GLSDGEWQQVLNVWGK) (Fig. 3A) and from this peptide labeled with RapiFluor (Fig. 3B) or 2-PCA (Fig. 3C). The HCD MS/MS fragmentation properties of the RapiFluor-labeled peptide appear to be rather unfavorable. The linkage between the RapiFluor moiety and the N-terminal amine is labile, resulting in intense signals at m/z 313 and m/z 240, which can be assigned to the intact RapiFluor molecule and to a fragment resulting from the loss of diethylamine. The intensity of sequence-specific peptide fragment ions is reduced compared to the spectrum of the unmodified peptide, which complicates peptide identification. Mainly for this reason, this derivatization method was not applied in subsequent experiments.

In contrast, the peptide modification with 2-PCA is largely stable in the gas phase. The N-terminal b ions are significantly enhanced compared with the spectrum of the unmodified peptide, probably due to the basic and easily protonatable pyridine ring of the 2-PCA modification. However, because of its relatively inefficient reactions and the heterogeneity of the reaction products, the 2-PCA derivatization method was not chosen for further experiments, either. The specific reactivity 2-PCA may be of interest in special cases, depending on the presence of lysine residues near the protein N-terminus.

### Derivatization of all free amino groups of proteins

Next, two well-established protein labeling reagents, tandem mass tag (TMT) reagents (Fig. 1E) and nicotinic acid NHS ester (Fig. 1F), were utilized to label both the N-terminal α-amino group of proteins and the ε-amino group of lysine residues. The NHS ester groups of these reagents primarily react with free amino groups of proteins. Mainly BSA and in addition myoglobin, β-lactoglobulin, α-casein, and β-galactosidase were used as model proteins to test these reagents and to optimize protocols. TMT reagents were designed for quantitative MS-based proteomics experiments [29]. They consist of an amine-reactive NHS ester group, of a spacer, and of a mass reporter. TMT reagents have been applied previously to N-terminal labeling of proteins in combination with quantitative analyses [41], and optimized peptide and protein labeling protocols are available. Our preliminary results confirmed that nearly complete TMT labeling of protein amino groups occurred. However, minor side reactions with tyrosine, serine, and threonine were detected, in accordance with earlier reports [42, 43]. These modifications may be misleading if they are incorrectly assigned to N-terminal amines of peptides.

**Fig. 4:**
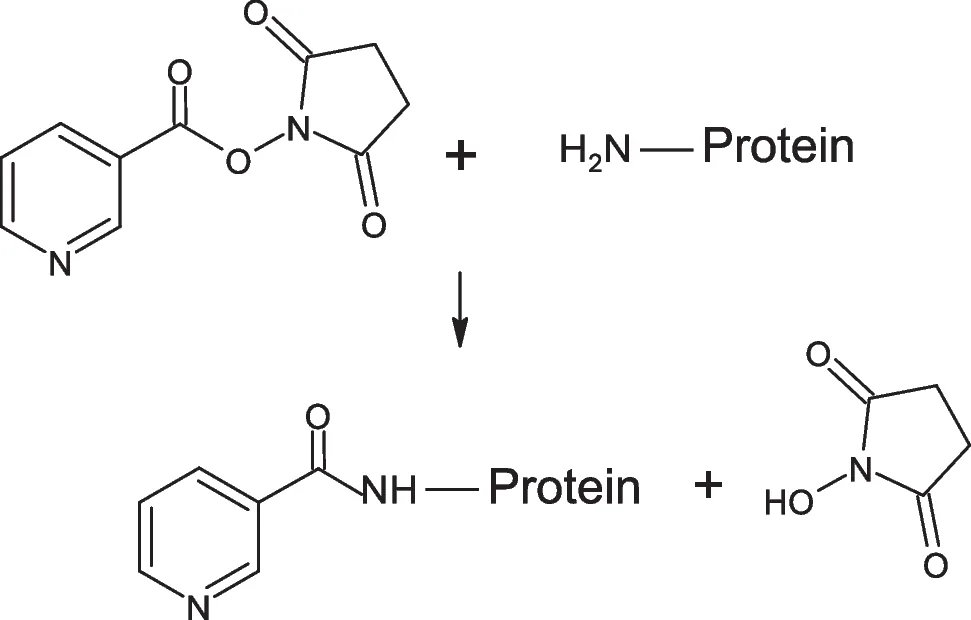
Reaction of nicotinic acid NHS ester with the N-terminal amino group of a protein

The derivatization of peptides or proteins with nicotinic acid NHS ester was originally introduced to facilitate de novo sequencing of peptides [30] and to allow relative quantification of peptides and proteins [30, 31]. For applications in quantitative analysis, reagents containing light or heavy isotopes were utilized. A reaction scheme for the chemical modification of the N-terminal amino group of a protein with nicotinic acid NHS ester is shown in Fig. 4. Due to the reactive NHS ester group, the reagent is largely selective for free amino groups of proteins and enables nearly quantitative derivatization. The basic nature of the nicotinic acid modification of peptides provides increased MS signal intensity, favorable MS fragmentation behavior, and high sequence coverage [30, 31]. Our initial nicotinoylation experiment using BSA as model protein and 20 mM nicotinic acid NHS ester reagent achieved an average labeling yield of 92% for the N-terminal peptide of BSA, which is comparable to the reductive dimethylation experiments. Side reactions of the hydroxy amino acids serine, threonine, or tyrosine rarely occurred.

**Fig. 5:**
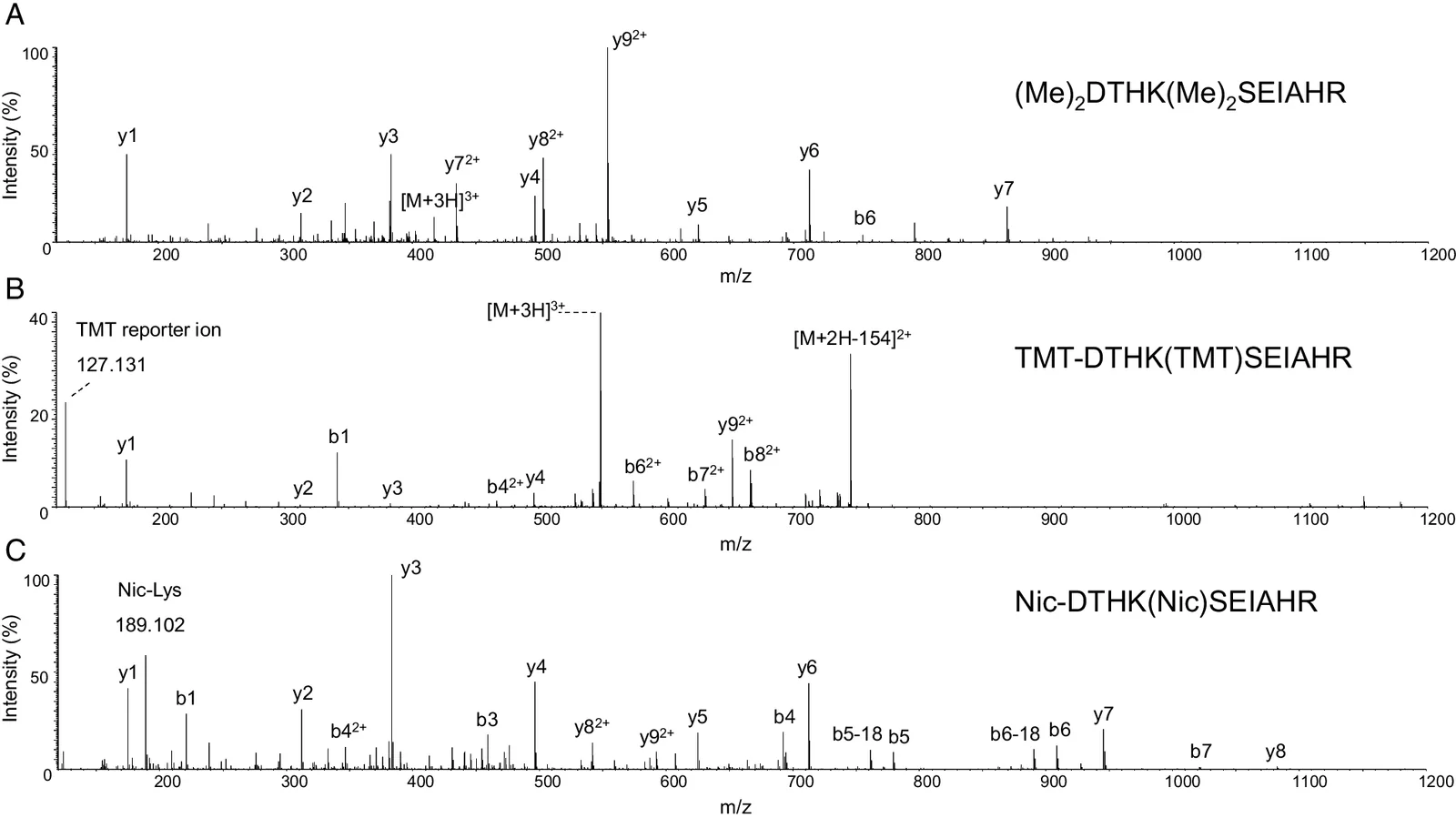
MS/MS fragmentation patterns of the N-terminal peptide of BSA (DTHKSEIAHR) with the following modifications at the N-terminal and the lysine amino groups: **A** Dimethylation, **B** TMT2-127 label, and **C** nicotinoylation

To characterize and compare the gas-phase fragmentation properties of the derivatized peptides, MS/MS data were acquired from the dimethylated N-terminal peptide of BSA (Fig. 5A), and from this peptide modified with TMT reagent (Fig. 5B) or with nicotinic acid (Fig. 5C). The spectrum of the TMT-labeled peptide shows an intense signal of the TMT reporter ion at m/z 127. An additional signal corresponding to the peptide ion that has lost a TMT-related neutral fragment of 154 Da dominates the spectrum. Unstable peptide modifications are unfavorable for the identification of peptides because they complicate spectra and decrease signal intensities of the sequence-specific b and y ions. In contrast, the modified amino acids of the dimethylated and of the nicotinoylated peptides were stable under HCD MS/MS conditions (Fig. 5A and C). No fragment ions resulting from these peptide modifications appeared in the spectra. The intense fragment ion at m/z 189 in Fig. 5C is specific for nicotinoylated lysine and was assigned to the known low-mass ion of lysine at m/z 84 [44] modified with nicotinic acid. Interestingly, the nicotinic acid modification increased the relative intensity of N-terminal b ions including b_1_ ions in comparison to the dimethylated peptide, in agreement with previous reports [30, 31].

### Nicotinoylation of proteins in solution

Based on the results from the initial experiments and on the gas-phase fragmentation properties of the modified peptides, we decided to focus on the nicotinic acid derivatization method. Critical experimental conditions were optimized to achieve more efficient protein labeling by nicotinic acid NHS ester, and to avoid misleading N-terminal labeling of internal proteolytic peptides by better inactivation and removal of the reagent prior to the proteolytic digestion.

The initial nicotinic acid NHS ester labeling protocol is described in the methods section. To optimize the pH, the nicotinic acid labeling reaction was performed in the range from pH 6.5 to 8.5 (Table 2). NHS esters have a limited lifetime in aqueous solutions, particularly at pH values above 8. A recent study [43] revealed that a relatively low pH value of 6.7 largely suppressed undesired side reactions of TMT reagents with the hydroxyl groups of serine, threonine or tyrosine. However, the reaction rates with amines will be slower at lower pH values. We obtained the highest reaction yield at an intermediate pH value of 7.5, which was chosen as the optimal pH for all subsequent experiments.

**Table 2:** Nicotinic acid NHS ester (10 mM) derivatization of the N-terminal peptide of BSA, DTHKSEIAHR, at five different pH values (6.5–8.5)

pH	Buffer (200 mM)	Doubly modified N-terminal BSA peptide (%)	Standard deviation (%) *n* = 2
6.5	MES	67	2.1
7	Sodium phosphate	67	5.0
7.5	Sodium phosphate	71	2.8
8	Sodium phosphate	68	2.1
8.5	TEAB	49	2.8

To further improve the reaction yield, the concentration of the nicotinic acid NHS ester reagent was increased from 10 to 20 mM (Table 3). For this and all following experiments, disulfide bonds were reduced by using tris(2-carboxyethyl) phosphine, but the resulting free cysteines were not alkylated, to avoid the presence of too many reagents during the nicotinoylation reaction. HEPES buffer at pH 7.5 turned out to be clearly superior compared to phosphate buffer at the same pH for this reaction (Table 3). An increase of the reaction temperature from 25 °C to 37 °C enhanced the reaction yield only slightly, so we continued performing the reaction at room temperature. The addition of guanidine hydrochloride was found to be the most important single factor to increase labeling efficiency, presumably due to protein denaturation and improved accessibility of amino groups.

**Table 3:** Optimization of the conditions for the derivatization of the N-terminal BSA peptide, DTHKSEIAHR, using 20 mM nicotinic acid NHS ester

Conditions	Buffer (200 mM) pH 7.5	Doubly modified N-terminal BSA peptide (%)	Standard deviation (%) *n* = 2
2 h, RT	Sodium phosphate	86	2.6
2 h, RT	HEPES	92	3.4
2 h, 37 °C	HEPES	94	0.6
2 h, RT	HEPES, 2 M guanidine	98	0.2

Finally, the reaction conditions for the inactivation of the nicotinic acid NHS ester reagent with hydroxylamine were optimized to prevent partial labeling of internal peptides during proteolytic digestion. The pH of the reaction solution was increased to 10 by the addition of ammonium hydroxide, which resulted in nearly complete quenching of the reagent. Free cysteines were extensively nicotinoylated, but the resulting thioesters were almost completely hydrolyzed by hydroxylamine. Furthermore, only very minor amounts of peptides containing nicotinoylated tyrosine, serine, or threonine were detected upon quenching.

The optimized procedure for the nicotinic acid derivatization was applied to BSA and to the additional model proteins myoglobin, α-casein, and β-galactosidase, to test broader applicability. The N-terminal peptides were released by trypsin or chymotrypsin cleavage. The reaction yields for N-terminal peptides of bovine serum albumin, myoglobin, and α-S1-casein were 98% or higher, and the yield for the β-galactosidase peptide was approximately 93% (Table 4).

**Table 4:** Percentage of complete nicotinic acid labeling of N-terminal peptides from test proteins in solution and in gel

Protein	Identified N-terminal peptide	Peptide labeling (%)	
		Solution	Gel
Bovine serum albumin	DTHKSEIAHR	98	84
Myoglobin	GLSDGEWQQVLNVWGKVEADIAGHGQEVLIR	99	
	GLSDGEWQQVL		92
α-S1-Casein	RPKHPIKHQGLPQEVLNENLLR	99	
	RPKHPIKHQGLPQEVL		92
β-Galactosidase	TMITDSLAVVLQR	93	76

The protein labeling efficiency did not appear to be heavily dependent on the N-terminal amino acid residues. The least complete reaction observed for β-galactosidase in solution may reflect incomplete denaturation of the large tetrameric protein even in the presence of 4 M guanidine hydrochloride.

### Comparison of protein nicotinoylation and protein dimethylation

Next, we compared the labeling efficiency of the optimized nicotinic acid labeling reaction to that of the dimethylation reaction. Dimethyl labeling of BSA was conducted in a mixture of 50 mM formaldehyde and 25 mM or 50 mM pyridine borane, and otherwise under comparable reaction conditions as the nicotinoylation reaction. The yield determined for the completely nicotinoylated N-terminal BSA peptide was 98%, with a standard deviation of 0.5% (*n* = 4), and the yield for the dimethylated N-terminal peptide was in the range of 95% ± 0.5%, irrespective of the pyridine borane concentration. Only undermethylated forms of the peptide, but no additional modifications caused by the reagents were considered. A potential advantage of the reductive methylation approach is the possibility to cleave proteins on the N-terminal side of dimethylated lysine by using the protease LysargiNase [45], facilitating detection of N-terminal peptides in certain cases. Even trypsin cleavages occasionally occur at dimethylated lysine residues [46].

### Nicotinic acid labeling of proteins in gel

N-terminal protein sequencing of in-gel samples is often required to identify N-termini of native proteins or of protein fragments. We adapted the nicotinic acid labeling protocol developed for proteins in solution to proteins in gel pieces. We found that complete removal of the chemicals in gels was required before protein labeling, and quenching and removal of the nicotinic acid NHS ester after the labeling reaction was essential, too. The amount of the reagent was increased from 20 to 40 mM to achieve a sufficiently high labeling reaction yield, and guanidine hydrochloride was omitted. Possible limitations of the in-gel workflow are proteolytic N-terminal peptides that are too short and hydrophilic or too long and hydrophobic to be recovered from gel pieces and detected by LC-MS/MS analysis. For this reason, proteins were cleaved not only with trypsin, but also with the complementary protease chymotrypsin in separate experiments. The reaction yields assessed for the N-terminal peptides of BSA, myoglobin, α-casein, and β-galactosidase were in the range from 76 to 92% (Table 4), and they were clearly sufficient for the identification of the N-terminal peptides in all cases.

## Conclusions

In summary, protein nicotinoylation represents a generally applicable derivatization method for N-terminal protein sequencing and compares favorably with the widely used reductive methylation and the other protein labeling approaches evaluated in this study. Nicotinoylated N-terminal peptides yielded informative tandem mass spectra with favorable fragmentation properties, facilitating their identification by LC-MS/MS analysis. Derivatization protocols using nicotinic acid NHS ester were optimized for N-terminal sequencing of proteins both in solution and in gel pieces. The nicotinoylation reaction yields were nearly complete or at least sufficiently high to enable identification of N-terminal peptides from all test proteins. In addition, protein nicotinoylation appears to be a promising derivatization strategy not only for purified proteins, but also for proteome-wide N-terminome analysis.

Although the nicotinic acid modification provided the most complete labeling of protein N-termini and represents our preferred approach, we consider protein dimethylation by formaldehyde and pyridine borane as a useful alternative workflow. The use of pyridine borane instead of the commonly applied reducing agent sodium cyanoborohydride is recommended, not only because of its lower toxicity, but also because of the slightly improved dimethylation efficiency observed in this study. Selective N-terminal labeling of proteins with 2-PCA followed by trypsin or Lys-C digestion at unmodified lysine residues appears to be advantageous only in specific applications.

## Data Availability

Data are available via ProteomeXchange with identifier PXD081064.

## References

[1] Rogers LD, Overall CM. Proteolytic post-translational modification of proteins: proteomic tools and methodology. Mol Cell Proteomics. 2013;12(12):3532–42. 10.1074/mcp.M113.031310. PMC3861706 23887885

[2] Varland S, Osberg C, Arnesen T. N-terminal modifications of cellular proteins: the enzymes involved, their substrate specificities and biological effects. Proteomics. 2015;15(14):2385–401. 10.1002/pmic.201400619. PMC4692089 25914051

[3] Lai ZW, Petrera A, Schilling O. Protein amino-terminal modifications and proteomic approaches for N-terminal profiling. Curr Opin Chem Biol. 2015;24:71–9. 10.1016/j.cbpa.2014.10.026. 25461725

[4] Edman P. A method for the determination of amino acid sequence in peptides. Arch Biochem. 1949;22(3):475. 18134557

[5] Koudelka T, Winkels K, Kaleja P, Tholey A. Shedding light on both ends: an update on analytical approaches for N- and C-terminomics. Biochim Biophys Acta Mol Cell Res. 2022;1869(1):119137. 10.1016/j.bbamcr.2021.119137. 34626679

[6] Kaushal P, Lee C. N-terminomics - its past and recent advancements. J Proteomics. 2021;233:104089. 10.1016/j.jprot.2020.104089. 33359939

[7] Chang E, Pourmal S, Zhou C, Kumar R, Teplova M, Pavletich NP, et al. N-terminal amino acid sequence determination of proteins by N-terminal dimethyl labeling: pitfalls and advantages when compared with Edman degradation sequence analysis. J Biomol Tech. 2016;27(2):61–74. 10.7171/jbt.16-2702-002. PMC4802820 27006647

[8] Vecchi MM, Xiao Y, Wen D. Identification and sequencing of N-terminal peptides in proteins by LC-fluorescence-MS/MS: an approach to replacement of the Edman degradation. Anal Chem. 2019;91(21):13591–600. 10.1021/acs.analchem.9b02754. 31573189

[9] Shen PT, Hsu JL, Chen SH. Dimethyl isotope-coded affinity selection for the analysis of free and blocked N-termini of proteins using LC-MS/MS. Anal Chem. 2007;79(24):9520–30. 10.1021/ac701678h. 18001127

[10] Kleifeld O, Doucet A, auf dem Keller U, Prudova A, Schilling O, Kainthan RK, et al. Isotopic labeling of terminal amines in complex samples identifies protein N-termini and protease cleavage products. Nat Biotechnol. 2010;28(3):281–8. 10.1038/nbt.1611. 20208520

[11] Hsu JL, Chen SH, Li DT, Shi FK. Enhanced a1 fragmentation for dimethylated proteins and its applications for N-terminal identification and comparative protein quantitation. J Proteome Res. 2007;6(6):2376–83. 10.1021/pr060639n. 17503795

[12] Means GE, Feeney RE. Reductive alkylation of amino groups in proteins. Biochemistry. 1968;7(6):2192–201. 10.1021/bi00846a023. 5690712

[13] Jentoft N, Dearborn DG. Labeling of proteins by reductive methylation using sodium cyanoborohydride. J Biol Chem. 1979;254(11):4359–65. 571437

[14] Weng SSH, Demir F, Ergin EK, Dirnberger S, Uzozie A, Tuscher D, et al. Sensitive determination of proteolytic proteoforms in limited microscale proteome samples. Mol Cell Proteomics. 2019;18(11):2335–47. 10.1074/mcp.TIR119.001560. PMC6823850 31471496

[15] Tamilselvan R, Dewes P, Cosenza-Contreras M, Huesgen PF. Hunter-Dia: An updated protocol for enrichment and mass spectrometry-based identification of protein N-terminal peptides. Methods Enzymol. 2025;719:25–42. 10.1016/bs.mie.2025.06.025. 40992842

[16] Hsu JL, Huang SY, Chow NH, Chen SH. Stable-isotope dimethyl labeling for quantitative proteomics. Anal Chem. 2003;75(24):6843–52. 10.1021/ac0348625. 14670044

[17] Kovanich D, Cappadona S, Raijmakers R, Mohammed S, Scholten A, Heck AJ. Applications of stable isotope dimethyl labeling in quantitative proteomics. Anal Bioanal Chem. 2012;404(4):991–1009. 10.1007/s00216-012-6070-z. 22644145

[18] Liu ZY, Zhou Y, Liu J, Chen J, Heck AJR, Wang FJ. Reductive methylation labeling, from quantitative to structural proteomics. TrAC Trends Anal Chem. 2019;118:771–8. 10.1016/j.trac.2019.07.009.

[19] Thielert M, Itang EC, Ammar C, Rosenberger FA, Bludau I, Schweizer L, et al. Robust dimethyl-based multiplex-DIA doubles single-cell proteome depth via a reference channel. Mol Syst Biol. 2023;19(9):e11503. 10.15252/msb.202211503. PMC10495816 37602975

[20] Hurtado Silva M, Berry IJ, Strange N, Djordjevic SP, Padula MP. Terminomics methodologies and the completeness of reductive dimethylation: a meta-analysis of publicly available datasets. Proteomes. 2019. 10.3390/proteomes7020011. PMC6631386 30934878

[21] Gidley MJ, Sanders JK. Reductive methylation of proteins with sodium cyanoborohydride. Identification, suppression and possible uses of N-cyanomethyl by-products. Biochem J. 1982;203(1):331–4. 10.1042/bj2030331. PMC1158228 7103947

[22] Metz B, Kersten GF, Hoogerhout P, Brugghe HF, Timmermans HA, de Jong A, et al. Identification of formaldehyde-induced modifications in proteins: reactions with model peptides. J Biol Chem. 2004;279(8):6235–43. 10.1074/jbc.M310752200. 14638685

[23] Liao R, Gao Y, Chen M, Li L, Hu X. A ubiquitous but overlooked side reaction in dimethyl labeling of peptides. Anal Chem. 2018;90(22):13533–40. 10.1021/acs.analchem.8b03570. 30372036

[24] Tayri-Wilk T, Slavin M, Zamel J, Blass A, Cohen S, Motzik A, et al. Mass spectrometry reveals the chemistry of formaldehyde cross-linking in structured proteins. Nat Commun. 2020;11(1):3128. 10.1038/s41467-020-16935-w. PMC7305180 32561732

[25] Cabacungan JC, Ahmed AI, Feeney RE. Amine boranes as alternative reducing agents for reductive alkylation of proteins. Anal Biochem. 1982;124(2):272–8. 10.1016/0003-2697(82)90038-0. 7149226

[26] Wong WS, Osuga DT, Feeney RE. Pyridine borane as a reducing agent for proteins. Anal Biochem. 1984;139(1):58–67. 10.1016/0003-2697(84)90388-9. 6430122

[27] Lauber MA, Yu YQ, Brousmiche DW, Hua Z, Koza SM, Magnelli P, et al. Rapid preparation of released N-glycans for HILIC analysis using a labeling reagent that facilitates sensitive fluorescence and ESI-MS detection. Anal Chem. 2015;87(10):5401–9. 10.1021/acs.analchem.5b00758. 25927596

[28] MacDonald JI, Munch HK, Moore T, Francis MB. One-step site-specific modification of native proteins with 2-pyridinecarboxyaldehydes. Nat Chem Biol. 2015;11(5):326–31. 10.1038/nchembio.1792. 25822913

[29] Thompson A, Schafer J, Kuhn K, Kienle S, Schwarz J, Schmidt G, et al. Tandem mass tags: a novel quantification strategy for comparative analysis of complex protein mixtures by MS/MS. Anal Chem. 2003;75(8):1895–904. 10.1021/ac0262560. 12713048

[30] Munchbach M, Quadroni M, Miotto G, James P. Quantitation and facilitated de novo sequencing of proteins by isotopic N-terminal labeling of peptides with a fragmentation-directing moiety. Anal Chem. 2000;72(17):4047–57. 10.1021/ac000265w. 10994964

[31] Schmidt A, Kellermann J, Lottspeich F. A novel strategy for quantitative proteomics using isotope-coded protein labels. Proteomics. 2005;5(1):4–15. 10.1002/pmic.200400873. 15602776

[32] Wisniewski JR, Zougman A, Nagaraj N, Mann M. Universal sample preparation method for proteome analysis. Nat Methods. 2009;6(5):359–62. 10.1038/nmeth.1322. 19377485

[33] Hughes CS, Moggridge S, Muller T, Sorensen PH, Morin GB, Krijgsveld J. Single-pot, solid-phase-enhanced sample preparation for proteomics experiments. Nat Protoc. 2019;14(1):68–85. 10.1038/s41596-018-0082-x. 30464214

[34] Turker C, Akal F, Schlapbach R. Life sciences data and application integration with B-fabric. J Integr Bioinform. 2011;8(2):159. 10.2390/biecoll-jib-2011-159. 21772064

[35] Perkins DN, Pappin DJC, Creasy DM, Cottrell JS. Probability-based protein identification by searching sequence databases using mass spectrometry data. Electrophoresis. 1999;20(18):3551–67. 10.1002/(Sici)1522-2683(19991201)20:18<3551::Aid-Elps3551>3.0.Co;2-2. 10612281

[36] Kulevich SE, Frey BL, Kreitinger G, Smith LM. Alkylating tryptic peptides to enhance electrospray ionization mass spectrometry analysis. Anal Chem. 2010;82(24):10135–42. 10.1021/ac1019792. PMC3075559 21114270

[37] Gurram S, Srivastava G, Badve V, Nandre V, Gundu S, Doshi P. Pyridine borane as alternative reducing agent to sodium cyanoborohydride for the PEGylation of L-asparaginase. Appl Biochem Biotechnol. 2022;194(2):827–47. 10.1007/s12010-021-03657-y. 34550501

[38] Griswold AR, Cifani P, Rao SD, Axelrod AJ, Miele MM, Hendrickson RC, et al. A chemical strategy for protease substrate profiling. Cell Chem Biol. 2019;26(6):901-907 e906. 10.1016/j.chembiol.2019.03.007. PMC6588500 31006619

[39] Bridge HN, Leiter W, Frazier CL, Weeks AM. An N terminomics toolbox combining 2-pyridinecarboxaldehyde probes and click chemistry for profiling protease specificity. Cell Chem Biol. 2024;31(3):534-549 e538. 10.1016/j.chembiol.2023.09.009. PMC10960722 37816350

[40] Song X, Ren X, Mei Q, Liu H, Huang H. Advancing in-depth N-terminomics detection with a cleavable 2-pyridinecarboxyaldehyde probe. J Am Chem Soc. 2024;146(10):6487–92. 10.1021/jacs.4c02222. 38421262

[41] Kalogeropoulos K, Bundgaard L, Auf dem Keller U. Sensitive and high-throughput exploration of protein N-termini by TMT-TAILS N-terminomics. Methods Mol Biol. 2023;2718:111–35. 10.1007/978-1-0716-3457-8_7. 37665457

[42] Wiktorowicz JE, English RD, Wu Z, Kurosky A. Model studies on iTRAQ modification of peptides: sequence-dependent reaction specificity. J Proteome Res. 2012;11(3):1512–20. 10.1021/pr2003165. PMC3310887 22335824

[43] Liao R, You P, Weng K, Li L, Song Y. TMT labeling under acidic pH overcomes detrimental overlabeling and improves peptide identification rates. Anal Chem. 2023;95(28):10595–602. 10.1021/acs.analchem.3c00525. 37403445

[44] Johnson RS, Biemann K. Computer program (SEQPEP) to aid in the interpretation of high-energy collision tandem mass spectra of peptides. Biomed Environ Mass Spectrom. 1989;18(11):945–57. 10.1002/bms.1200181102. 2620156

[45] Huesgen PF, Lange PF, Rogers LD, Solis N, Eckhard U, Kleifeld O, et al. LysargiNase mirrors trypsin for protein C-terminal and methylation-site identification. Nat Methods. 2015;12(1):55–8. 10.1038/nmeth.3177. 25419962

[46] Chen M, Zhang M, Zhai L, Hu H, Liu P, Tan M. Tryptic peptides bearing C-terminal dimethyllysine need to be considered during the analysis of lysine dimethylation in proteomic study. J Proteome Res. 2017;16(9):3460–9. 10.1021/acs.jproteome.7b00373. 28730820

